# Religious beliefs and climate change adaptation: A study of three rural South African communities

**DOI:** 10.4102/jamba.v10i1.509

**Published:** 2018-10-16

**Authors:** Simone Schuman, Jon-Vegard Dokken, Dewald van Niekerk, Ruth A. Loubser

**Affiliations:** 1 Unit for Environmental Sciences and Management, African Centre for Disaster Studies, North-West University, South Africa; 2 Department of Sociology and Human Geography, University of Oslo, Norway; 3 School of Philosophy, North-West University, South Africa

## Abstract

This article argues that religious beliefs significantly influence a community’s understanding and experience of climate change adaptation, indicating the need for an inclusion of such information in climate change adaptation education. Data were collected using the Q-method, whereby recurring statements were identified from semi-structured interviews with participants from three rural communities in the North-West province of South Africa: Ikageng, Ventersdorp and Jouberton. The research found that community members who regard themselves as religious (overall of the Christian faith) fall under two groups: the religious determinists or fatalists, who see climate as a natural process that is governed by God, and religious participants who deny this ‘naturalness’ and acknowledge humans’ impact on the climate.

## Introduction

Human behaviour is indisputably influenced by religious beliefs and religion (Hulme 2009; Slimak & Dietz 2006). Such behavioural influences include the way in which people perceive and interact with the physical environment. Climate change is widely considered as being ‘the most complex and serious environmental issue that human societies have ever faced’ (O’Brien, St. Clair & Kristoffersen 2010:3). Consensus between scientists and researchers alike is that Africa, with its many developing countries, will be most impacted by this global crisis (Mendelsohn 2007; Toulmin 2009; UNFCCC 2007). Adaptation and mitigation are crucial when it comes to managing the impacts of climate change. However, when it comes to climate change adaptation, a greater focus on the human dimension of climate change than on mitigation strategies is required. As Jenkins (2013:17) puts it, climate change threatens to ‘disinherit cultures of the concepts and practices that sustain a way of being human’; and therefore, changes in climate are inextricably linked to the social context within which they take place (Lorenzoni et al. 2000; O’Brien et al. 2010). This allows for the development and implementation of context-specific strategies where a community’s individual climate change issues and needs can be addressed (Richards et al. 2013:113), as part of the social context referred to by Lorenzoni et al. (2000) and O’Brien et al. (2010) concerns peoples’ beliefs.

Whether it is about humankind’s place in the universe or about which sports team is the best, beliefs span every aspect of people’s lives. However, Mbiti (1977:1) contends that it is the religious belief that permeates ‘into all the departments of life so fully that it is not easy or possible always to isolate’, therein lies the issue of defining religious beliefs; hence, for the purposes of this article, religious beliefs will be defined as being (Schilbrack 2013):

[…] composed of those social practices authorised by reference to a superempirical reality, that is, a reference to the character of the Gods, the will of the Supreme Being, the metaphysical nature of things, or the like. (p. 313)

The words ‘social practices’ in the definition above point to a vital component of what can be considered African traditional religions: the inextricable nature of African communities and their religion (Mbiti 1977:2). According to Mbiti (1977:15), for African people, religion is ‘an ontological phenomenon’ that ‘pertains to the question of existence and being’. Traditional African religions view nature not as a separate, impersonal object or phenomenon, but rather as being ‘filled with religious significance’ (Mbiti 1977:56). Many rituals, rites, ancestral beliefs and more in traditional African religions relate directly to nature (Olupona 2006:264). Consequently, it is not much of a leap to expect that these religious beliefs will influence a person’s adaptation to climate change.

Against this backdrop, the research presented in this article attempts to give a better idea of the communities’ understanding and experience of changing climate within their own specific context, in order to address the problem statement that religious beliefs significantly influence a community’s understanding and experience of climate change adaptation, indicating the need for an inclusion of such information in climate change adaptation education.

After a brief literature review on the link between religious beliefs and climate change adaptation follows a discussion of the study’s context, methodology and results. Based on these findings, this article concludes to show that the fundamental beliefs of community members, more specifically their religious beliefs, greatly influence both their perceptions of and adaptation to climate change. Finally, recommendations are made on the incorporation of community and individual religious beliefs when promoting or implementing adaptive strategies to alleviate the effects of climate change.

## Literature review

### Theory of religious beliefs and climate change adaptation

Determining what the stance is of the religions involved on the place of humankind in nature and what their views on ecology are, is prudent when investigating the influence that religious beliefs have on climate change adaptation. In support of this, although referring specifically to climate change risk, McNeeley and Lazrus (2014:506) state that ‘the way in which people perceive climate change risk is informed by their social interactions and cultural worldviews comprising fundamental beliefs about society and nature’. Hulme (2009) concurs that:

… our beliefs about the divine, about the spiritual and the transcendent, and about our role in the world as moral agents, shape our sense of duty and responsibility to care for others and for Nature. (p. 161)

Therefore, perceptions of climate change risk and vulnerability, along with religious conceptualisations of nature and the human-nature relationship, influence the feasibility and acceptability of adaptation planning, policy-making and implementation. For the purposes of this study, only Christianity and Islam, along with a broad overview of traditional African religions, are briefly considered. This is owing to the fact that both Christianity and Islam are identified as being prevalent, aside from any traditional African religions, on the African continent (Mbiti 1977; Turner 2010). The link between religion and climate change, and climate change adaptation are discussed in addition, although within a broader context of different religions.

### Humankind and nature: Views from Christianity, Islam and traditional African religions

Christianity and Islam are both what is referred to as Abrahamic religions, alongside Judaism. Within these religions, the significance of nature is not merely instrumental; nature forms part of God’s creation, and humankind’s relationship with it is a pragmatic one (Cooper & Palmer 1998:31). Christian doctrine, when describing humankind and nature, relies on two key concepts according to Davies (1994:31): relationship and responsibility. To Christians, the term *relationship* entails a direct connection with nature, where ‘distinctions between nature and culture are inappropriate’ (Davies 1994:31). The second, *responsibility*, ‘involves moral issues’ (Davies 1994:31) and includes the stewardship over nature that God gives humankind. This stewardship, however, can be interpreted in two very different ways. On the one hand, it implies humankind’s dominion over nature, using it for their own gain without giving much thought to the consequences (Edwards 2007; Hulme 2009). On the other hand is the interpretation of caretaker, where humans are appointed as nature’s guardians and are appointed to take care of Earth’s resources (Russell 1994:147). The distinction between these two interpretations underpins the link between religious beliefs and climate. Hence, two diverging views on adaptation may arise from these two interpretations of the Christian stewardship of nature.

Although it shares many similarities with Christianity, Islam has a slightly different view on nature. Islam’s main concern is obedience to God and the penalties for disobedience (Forward & Alam 1994:79). This extends to the relationship between humans and nature, as the religion ‘warns of the consequences of disobedience in human plunder and exploitation of the planet earth’ (Forward & Alam 1994:79). An important distinguishing aspect, however, is the view that creation will be destroyed once God takes his faithful servants to Paradise; the function of our world is merely to provide an arena for God’s interaction with humans (Forward & Alam 1994:79). To them the world that they inhabit is perishable, and there is no reason to safeguard or protect it (Forward & Alam 1994:98). This particular view does not bode well for the possibility of adapting to climate change.

As emphasised, humanity and religion are interwoven, implying that natural phenomena, and indeed nature in itself, inextricably form part of traditional African religious beliefs (Mbiti 1977:48). Traditional African religions are based on the philosophical foundation of what Turaki (1999:97–98) refers to as the ‘Law of Harmony’ – within this foundation, ‘man stands face to face with the “physical”, the “material” and the “spiritual” dimensions of his world’ (Mbiti 1977; Turaki 1999). This illustrates the anthropocentric nature of African ontology, where the dictating view is that humankind is placed at the centre of the created universe (Mbiti 1977:48). Here nature is placed within the realm of the spirit beings and the impersonal powers that govern the world which, although seen as a holistic part of creation, is created by the Supreme Being (God) (Turaki 1999:98). This interplay between the spirit and the physical worlds is described by Mbiti (1977:57): ‘The invisible world is symbolised or manifested by these visible and concrete phenomena and objects of nature’. Furthermore, the relationship with nature is defined in terms of communality – community represents an important part of the traditional African religious philosophy and entails humankind’s relationship with nature as well as humanity. In fact, ‘man (sic) is not independent, but dependant’ (Turaki 1999:101).

Throughout three religious frameworks discussed above, one main aspect becomes apparent: nature was created by God, or a higher power, and humankind stands at the centre. The potential implication of this shared belief is that willingness to adapt can go either way – whether a person adapts to climate change or cannot, will be influenced by the meaning they associate with humankind’s relationship to God’s creation. In fact, Hulme (2009:144) states that one of the reasons people disagree about climate change is that ‘[they] believe in different things about [their] duty to others, to Nature and to [their] deities’.

### Religion, climate change and climate change adaptation

According to McCown (1927:521), the link between religion and climate may be more pronounced than generally realised. Decades after him, Veldman, Szasz and Haluza-DeLay (2013:3) echo other authors in asserting that religions may become critical agents in the fight against climate change. Although multitudinous in nature and in their way of exerting influence, world religions have the ability to ‘decisively impact how societies all over the world respond’ to climate change (Veldman et al. 2013:3). In recent years, various studies have specifically highlighted the link between religion (and with it religious beliefs) and climate change (and with it climate change adaptation) (e.g. Bergmann 2009; Brownlee, Powell & Hallo 2013; Gifford 2011; Hulme 2009; McCown 1927; McNeeley & Lazrus 2014; Nagle 2008; Richards et al. 2013; Stern et al. 1999). This section briefly summarises relevant extant literature on the subject.

Along with the cultural implications posed by climate change as put forth by Jenkins (2013:17), Bergmann (2009:98) maintains that ‘climate change challenges and changes images of God and the sacred and their corresponding sociocultural practices’. These challenges include the possible necessary adaptation of religious practices, and indeed beliefs, to face the growing global climate crisis. Some of these practices are, for instance, related to myths, based in traditional African religions, which have been and continue to be contributing factors to climate change (Waapela 2016:8). Still there are positive influences to be received from religion’s influence on climate change adaptation.

Pro-environmental religious beliefs have been shown to associate directly to the willingness and capacity to adapt to climate change; in a study conducted on Pacific Island countries, Nunn et al. (2016:14–15) found that the high degree of spiritual engagement with nature (based on religious beliefs) creates possibilities for the communication of adaptive measures to these communities. Furthermore, Nunn et al. (2016:477) assert that if these communications are made through religious channels, rather than secular ones, the reception will be more positive.

Nagle (2008:69) identified two distinctly opposing views within the group of American Evangelicals regarding what should be done about the climate crisis: firstly, there is the belief that adaptation needs to take place to preserve the earth, and secondly, the belief that the earth is able to restore itself. Both these beliefs, however, are constituents of the overarching belief that God created a ‘good’ earth (Nagle 2008:69); humankind’s intervention in its survival is the only point that is disputed. Even so, scepticism towards climate change remains a reality within this religious group. Research conducted by Veldman (2016:212) clearly points to Evangelicals’ ‘unique religious outlook’ (regardless of what that may entail) as being a possible determinant of the climate change scepticism prevalent within this religious group. This brings us to the other side of the religion–climate change coin: the negative influences of religious beliefs.

Although seemingly a likely tool for achieving adaptation goals, religion is also often cited as one of the many limitations to climate change adaptation. Taoism can be used as an illustration of how religion can hamper adaptation to climate change if it is taught and not already implemented through individual decision. Within the Taoist belief, ‘to contend with other people, to try to push them about, to try to mould, educate, and refine them’ will lead to some problems (Kinsley 1995:79). Based on this thinking, attempting to implement climate change adaptation, or even attempting education on the value thereof, may be met with some resistance within the Taoist community, simply because of their religious beliefs.

In their study on climate change perceptions among rural farmers, Cullen and Anderson (2016:11) found that the self-proclaimed religiosity (Catholicism) of a quarter of their participants had a significant influence on their environmental concern: participants who identified themselves as Catholics were found to have lower levels of concern for the effects of climate change on food production.

Yet, this dichotomous nature of the link between religion and climate change adaptation is not the point to be taken from literature on the subject; what lead to the undertaking of this study is in fact the evident influence that religion appears to have on adaptation, whether positive or negative. Hence, the initial aim was to establish the presence of this link within the specific context of the study area. Veldman et al. (2013:4) and Hulme (2009:144) stress the importance of remembering that the way in which religion engages with climate change in one locale can differ significantly to the way it does in another. In fact, it is for this very reason that this study is important: research on religion and climate change needs to be context-specific if the overall aim is to improve the adoption of adaptation measures. This study’s secondary aim is to provide conclusions pertaining to the positive or negative nature of the link between climate change adaptation and religious beliefs.

### Study area: Three rural communities in North-West province, South Africa

The three communities, Jouberton, Ikageng and Ventersdorp, of the North-West province are predominantly of the Setswana culture and language. During the data collection process, 11 participants who identified themselves as being affiliated with a specific religion indicated that they were Christians, with elements of traditional African religions present. This affirmed the researchers’ expectations regarding the religious affiliations, namely possible connections between these communities’ rural statuses and possible subsequent traditional worldviews.

These communities were further chosen because of the fact that this study forms part of a larger project, and therefore, the researcher was confident that the communities met the necessary requirements. This overarching project was undertaken in conjunction with the South Africa–Norway Research Co-operation (SANCOOP) and centres on belief systems and climate change adaptation. Three separate studies, including this study, were conducted under this project and covered different aspects relating to the link between overall belief systems and climate change adaptation.

## Materials and methods

### Research design

As this study involved people’s personal experiences regarding climate change adaptation and religious beliefs, as well as determining specific worldviews, the qualiquantological approach of Q-methodology (Stenner & Rogers 2004) was deemed the most appropriate research design. This methodology was used to determine the communities’ experiences and understandings of climate change adaptation while producing statistical data to confirm, as based on literature (e.g. Bergmann 2009; Brownlee et al. 2013; Gifford 2011; Hulme 2009; McCown 1927; McNeeley & Lazrus 2014; Nagle 2008; Richards et al. 2013; Stern et al. 1999), that there is a link between climate change adaptation and people’s religious beliefs, and to create a series of worldviews regarding climate and beliefs with which participants could identify. Q-methodology is especially suited to investigate the influence of religious beliefs on people’s willingness or ability to adapt to climate change, as it enables the researcher to quantify and illustrate abstract concepts such as beliefs and find correlations between different subjects, in this case religious beliefs and climate change. Previte, Pini and Haslam-McKenzie (2007:141) maintain that Q-methodology allows for a ‘focus on the subjective experiences of participants’ and an ‘emphasis on context’, making it a well-suited option for this study as it investigated specifically individual and communal participant views and beliefs. In turn, it was hypothesised that these beliefs would influence climate change adaptation, further strengthening the case for using Q-methodology for this study.

Q-methodology addresses the problem of finding a scientific methodology with which to study subjective phenomena such as opinions or, in this case, fundamental beliefs (Previte et al. 2007:136).

The research comprised four phases. Phase One consisted of semi-structured interviews, from which 40 significant statements (Q-sorts) were identified for use in further phases. During phases Two and Three, participants were asked to rate these statements on a Likert-type scale in order to determine the level of agreement and disagreement. Phase Four involved participants identifying one of the five worldviews (compiled from Phase Two and Phase Three data) that they identified with most.

### Sampling

Three rural communities in the North-West province of South Africa were chosen for the collection of data: Ikageng, Jouberton and Ventersdorp. Respondents were chosen using purposive sampling, based on their willingness to participate. Furthermore, snowball sampling was used to find additional participants when initial sampling attempts were unsuccessful.

A total of 103 participants were involved in Phase One of the research, consisting of men and women between the ages of 18 and 59; 51 of these participants were invited to take part in Phase Two based on a random selection and participant availability, and 25 in Phase Three. Phase Four had a total of eight participants, chosen based on their significant loading for each of the factor narratives (worldviews).

The study’s overall sample was kept relatively small in order to keep with Q-methodology requirements: Q-methodology is used to obtain and analyse individual views and is not suited to generalisation. This method was ideal for this study, with which the researchers wanted to motivate the importance of the unique, individual voice within the community, while keeping in mind the larger community context.

### Instrumentation

This study utilised the Q-method for data collection.^1^ It was chosen specifically for its capacity to produce statistical data derived from qualitative input. The Q-methodology’s combination of qualitative and quantitative processes enabled the systematic identification of participants’ subjective experiences regarding religious beliefs and climate change adaptation, ultimately leading to the creation of specific narratives on the topic.

### Data collection

Data collection during Phase One consisted of semi-structured individual interviews. Two questions were asked: (1) ‘*what* do you think about the climate?’ and (2) ‘*do* you think it would be possible to change your beliefs about the climate?’ Probing questions were asked throughout to ensure that detailed answers were provided for the collection of rich data. Interview transcripts (translated from Tswana and Afrikaans to English where applicable by mother tongue speakers) were analysed and 40 significant statements (referred to as Q-sorts) regarding the climate and beliefs were identified.^2^

During Phase Two, participants were instructed to rank all 40 statements on a Likert-type scale based on the extent to which they agreed or disagreed. This was done using specially prepared whiteboards and magnetic statement cards. The Likert scale ranges are: strongly agree (+3), agree (+2), slightly agree (+1), neutral (0), slightly disagree (-1), disagree (-2) and strongly disagree (-3). A seven-point scale was utilised in order to allow for a greater variety of options and nuances in terms of the differences between beliefs in the presented statements. Instructions were limited, and participants were free to rank statements at their own discretion, creating a free distribution of data along seven different rankings.

In Phase Three, participants were given the same board and statements, but they were instructed to rank a predetermined number of statements per category or rank, thereby ensuring a forced distribution of data. Therefore, participants were no longer at liberty to rank statements at their own discretion – they were instructed in the number of statements that could be placed under each ranking, forcing the distribution.

Finally, in Phase Four, statistically correlating statements were grouped to create five different narratives (factors). Analysis with the Q-method is mostly done using specific computer software, called PQMethod, that calculates the level of agreement and/or disagreement between individual Q-sorters, groups sorts together based on similarities or dissimilarities, calculates the factor scores of each Q-sort and finally describes and interprets the factors (Van Exel & De Graaf 2005:8–10) to create these narratives. Eight participants from Phase Three were identified as loading significantly for the various narratives and were asked to return for the final research phase. The eight participants were presented with the five narratives (worldviews) and were requested to choose the one that they related to the most. Audio recordings were made of all the interviews conducted, throughout each of the four phases, to allow for qualitative analysis of participants’ statements. Study phases took place 2–4 weeks apart.

### Data analysis

A literature review of the relevant concepts provided a sound theoretical foundation upon which the study could be based. Thereafter, a qualitative analysis of the participant interviews was undertaken, during which statements regarding beliefs in terms of climate, climate change and religion were identified and analysed, in addition to the analysis done with the Q-method, to obtain statistical data out of the Q-sort and to factor narrative feedback from participants. Computer software used in the analysis for phases Two to Four calculated the level of agreement and/or disagreement between individual Q-sorts, grouped sorts together based on similarities or dissimilarities, calculated the factor scores of each Q-sort and finally provided a description and interpretation of said factors (Van Exel & De Graaf 2005:8–10). Principal component analysis (PCA) was used throughout the analysis process.

## Results and discussion

This section outlines and discusses the overall results obtained from the study. Coding for Phase One to Phase Three participants is based on the audio files of the interviews (e.g. 01-GP-Ikageng-14.13^3^), whereas Phase Four participant coding is based on the order in which interviews were conducted in that phase (e.g. Participant #1).

### Qualitative data

Owing to the complex and often personal nature of religious beliefs, much of the qualitative data obtained from the interviews during each of the four phases were used. It was theorised, correctly, that many of the interesting results obtained through the Q-sorts and subsequent worldview interviews in phases Two to Four might be better interpreted and supported by the qualitative statements made by participants before and during these phases. Therefore, rather than relying solely on the factor results, this study contained a strong qualitative component.

The following section discusses the qualitative findings from Phase One. Additionally, links between the qualitative data and the Q-sort data are addressed in subsequent sections.

#### Phase One: Qualitative data

Participants were free to attribute any meaning of their choice to the terminology used in the open-ended questions asked in Phase One. First important observation was that the term ‘beliefs’ was often immediately associated with religious beliefs:

‘For me? You know when you talk about belief, I automatically think whether you’re Christian – religious – whether you’re religious or you believe in a higher God or you believe in witchcraft and whether those things affect the way that you think about the climate.’ (04-SS-Ikageng-7:11)

It is important to note that although participants who identified themselves as religious indicated that they are Christians, no denominations were specified. Beliefs in the mystical powers of traditional healers, as well as rituals, were expressed, indicating ties to traditional African religions, although many participants who identified themselves as Christians refuted these beliefs. This is interpreted as religious exclusivity and is a common characteristic of many religious systems.

Certain participants indicated that they believe that the climate was created by, and is also controlled by, God:

‘It’s – Weather is like that, today is cold and it cannot change. It’s God who created it.’ (08-KM-Ikageng-03:00)

‘Climate is controlled by God, God created all things in the world.’ (02-KM-Venterdorp-7:46)

Others opted for stating that the climate is ‘natural’ or part of nature:

‘I don’t think it would be possible to change my beliefs about the climate because every single weather condition is natural and there’s nothing we can do. If it’s raining then there’s nothing you can do about it, you can’t change rain.’ (06-KM-Ikageng-3:22)

At least two participants, however, attributed both these qualities to the concept of climate; they indicated that to them the climate is natural and that that inherently means that it was created by God, and vice versa:

‘It’s a natural thing, something we are born into. When it rains, I as person do not have the ability to stop the rain it’s God’s will.’ (05-KM-Ikageng-4:38)

‘Yes. So I don’t have that belief I can change the climate because of it’s natural.’ (03-SS-Ikageng-6.22) (when asked if the climate is God’s thing)

The extent to which religious beliefs influence a person’s capacity or willingness to adapt to climate change is demonstrated in the following statement (who just before expressed that they believed the climate is ‘God’s thing’):

‘I can’t change anything. Because it is the thing that we got in the world. That’s why we can’t change anything, and I don’t have a belief so that it can change or what.’ (03-SS-Ikageng-6:22)

This statement lays the foundation for this study’s assertion that religious beliefs have an influence on climate change adaptation. Evidently, the belief in a God who created the climate along with the rest of the earth prevents the participant from believing that they can have any effect on or bring about any change to the climate. This kind of assertion indicates a type of determinism, without the associated doom-and-gloom, regarding humans’ impact on the climate, which explains their probable indisposition to adapt to its change. The following statement can be evaluated similarly:

‘How I believe, for me my perspective cannot change what already God has decided. I don’t know, maybe you help me in that, because you studied this, I didn’t study it, but how I think is how God is created things and it should be like that. My culture cannot change the way God has created.’ (02-BJ-Ventersdorp-16:02)

The participant above also made no mention of humans causing climate change or even contributing to it in any way – this enforces their belief that climate change is God’s will.

Another type of determinism presented a more fatalistic, metaphorical ‘shrugging of the shoulders’ manner:

‘I just have to accept only God knows because I think he is the one doing this, and I think we can blame it on the kids for not listening perhaps God is punishing us because we can’t always blame the change in weather. God put us here on earth we just have to accept the way things are, the way they are going whether he’s punishing us because of our kids’ engagements, we don’t know. Sometimes he just scares us and we just have to accept.’ (04-GP-Ventersdorp-6:16)

Another prominent theme is illustrated in the statement above is this: climate change is punishment from God:

‘I think that all this things are caused by God, God is punishing us.’ (07-RM-Ikageng-3:34)

The reasons for this punishment vary, which include the following:

- Abortion:
  ‘Climate it’s because of people that get the abortion, it’s because God he don’t like it’, cause we kill babies’ spirit, where they are unborn because there is a people that they don’t have a kids, then God he gives you a baby and then you will kill. No matter its three days. The womb it’s already touched then other people that will become it, touched. So I’m thinking it’s punishment.’ (02-AB-Jouberton-16:56)
- Violence and murder:
  ‘Remember God said “do not kill”, when you walk at night even if is not so dark when going to the shops you will meet criminals and they will search you and take your money and if they know you, they will stab you and kill. That is the first punishment of God.’ (07-RM-Ikageng-3:34)
- The ‘sins of the father’ – sins committed by previous generations:
  ‘Second punishment of God is that I will punish new generation based on old’s [sic] generations sins.’ (07-RM-Ikageng-3:34)

Failure to change the causal behaviour in response to punishment results in further punishment, therein lies the potential for this religious belief to assist adaptive efforts.

Climate change was also attributed to signs of the Apocalypse, or the end of the world, citing the Bible as proof:

‘According to my belief. These things that are happening currently, it’s like the prophecies in the Bible, not from someone, but from the Bible are coming true. They are coming true have you noticed?’ (01-GP-Ikageng-14:13)

The above statement refers to biblical prophecies heralding the second coming of Christ, and the signs of the end of the world, one of which the participant identifies as air pollution (a contributor to climate change).

A synthesis of biblical Christianity and traditional African ancestral belief reflected in the Phase One interviews:

‘The Bible said nè, you can’t blame me, you can’t blame me because if you don’t know my picture of me, because of – you don’t know me, why you blame me, maar you can’t blame your next neighbour. You see, at least you must like your neighbour before you can like me. So it’s just that we can … no one have …. I have seen the Lord. We dream of ancestors, we dream of all people that died. We see that. So He uses ancestors.’ (02-AB-Jouberton-16:56)

This participant maintained that it is indeed the ancestors causing climate change, but that they are ultimately God’s vehicles to punishment. Ancestors are regarded as intermediaries between humans and God (McVeigh 1974:35).

Another intriguing aspect of African religions and tradition is the belief that traditional healers possess otherworldly, even magical powers inherent in nature which they can manipulate. Although most participants who made mention of these traditional healers ultimately denied any belief in the truth of their powers, some participants did indicate that they believe that traditional healers have the power to alter the climate:

‘Yeah, we believe in them. That a traditional doctor has struck somebody with lightning, the traditional doctor has made it rain. I mean they do indeed work for us, so?’ (02-GP-Ikageng-4:55)

The use of rituals to bring about desired changes in the climate was mentioned by some participants, whether it was only to illustrate some of their culture’s traditions or to state their own beliefs in their power. For instance, these rituals include women in a community who have lost children, coming together to ‘wash off the blood of their children’, after which this water is poured out all across the community. This is believed to bring rain (01-SS-Ventersdorp-20.00).

Upon encountering more education and scientific knowledge on the topic of climate change, some participants felt that they would be able to adapt specific religious beliefs they held accordingly. They proposed religious institutions as bases of operation for raising climate change awareness and in so doing, highlighted the importance of these institutions in encouraging community participation:

‘Ja, if we get taught every time about climate change, like there’s projects where people tell people if we do something like this which is wrong that it will affect climate change in a certain way, like at schools, maybe at church, at the house, maybe do some projects just to show people that something that we do can affect climate change in a certain way and how we will be affected also as people.’ (05-BJ-Ikageng-5:58)

Others held this education to be contrary to religion; for them, education discredits religious belief:

‘I don’t think--well now, it’s way different, you know, but there are still those people that believe that it’s not really the climate; it’s something to do with the gods, or whatever, not being happy with us, and that kinda stuff you know. But personally, I don’t believe that. I think I have enough information to know it’s not about that.’ (04-SS-Ikageng-7:11)

‘… I don’t believe in religious things, I believe in scientific theories …’ (01-KM-Ikageng-8:10)

### Phase One: Conclusion

Participants indicated overall awareness of changes in climate and attributed these changes to various causal factors. Statements such as those in the previous section were used to compile the Q-sort set of 40 statements which was used in subsequent phases. Findings from these phases are discussed in the following sections.

### Q-sort results: The five factors (worldviews)

This section summarises the results of the overall Q-sort process, including the five factors that were identified and used in Phase Four.

According to the analysis, the five factors (worldviews) accounted for 58% of the sample variance, with the religion-related factors 2 and 3 accounting for 32%. Table 1 describes each worldview and refers to each by its descriptive title. Table 2 contains Phase Four participants’ significant factors, as well as their chosen worldviews.

**TABLE 1:** Factors and their descriptions.

Factor (worldview)	Description and/or factor narrative
Factor 1: Collectivist and/or liberal	‘The climate change we experience today is not a punishment for people’s sins and neither is it a sign that the world is ending, but rather a natural occurrence where nature wants to reshape itself. Since the climate affects how people’s emotions, it can also cause people to change their beliefs. If we unite we have better chance at solving climate problems and influencing the next generation’s attitudes towards nature. This is important since the climate influences the growth of crops and production of food, and we have to act now to prevent further changes to the climate’.
Factor 2: Religious determinist and/or fatalist	‘The climate can’t be changed by traditional healers since it’s determined by God. Because of this, the climate is just a natural part of the world that we have to accept and it is not affected by people’s behaviour. Climate change is not related to the burning of fossil fuels, climate is just unpredictable. I’m open to changing my beliefs, but I think the best way of solving any possible environmental problems is by returning to the ways of our ancestors. Things were better when I was younger, and I think that today’s technology plays an important part in the changes we see. In others words; I don’t believe that there is anything wrong with the climate and I don’t think environmental problems are a sign that the world is ending’.
Factor 3: Religious	‘The climate is determined by God and climate change is a sign that the world is ending. I will not change my belief and the climate influences neither me nor other people emotionally. The climate is not that complicated seeing as the changes are mainly related to the burning of fossil fuels. This means that we can also control the climate through technology, and that natural disasters are largely caused by people’s actions. The next generation will be influenced by our behaviour towards nature, but that doesn’t mean we should return to the ways of old. The climate was not better when I was younger, and I think young people can help the older generations get educated about climate change. This is important, since we have to act now to hinder further damage to the environment’.
Factor 4: Technology and/or human	‘The climate plays an important part in our lives and we need to respect the environment. We have the right to know about climate issues that affect us directly and indirectly. Even though the climate is changing, it’s not caused by population growth and is not a sign that the world is ending. Sometimes I think that traditional healers can cause the climate to change, but I also believe that the changes in the climate are related to the burning of fossil fuels and people damaging the environment when they are trying to make money. This means that we should rather try to use sustainable technologies, since this would benefit the environment. It may not be possible for humans to control the climate through technology, but if we work together, we can make a difference’.
Factor 5: Governance and/or structural	‘There is something wrong with the environment, but returning to our old ways is not the way to solve problems. Seeing how the climate is both complicated and unpredictable, the government plays an important part in informing people about the changing and drafting laws as an effective way of protecting the environment. There is no way around the fact that the changes we see today are consequences of people’s behaviour, and if we continue the way we are now we will destroy the earth. However, it might be difficult to educate people about the problems because of their beliefs, but I for one am open to changing what I believe to be true’.

**TABLE 2:** Phase Four: Participants’ significant factors and chosen worldviews (as clarified in Table 1).

Factor	Participant no.	Chosen worldview
1	1	5
2	2	1
3	3	1
	4	5
4	5	4
	6	1
5	7	4
	8	4

It was interesting to note that neither of the two worldviews that are geared toward religious beliefs (factors 2 and 3) were chosen, although qualitative statements were made during the Phase Four interviews that could suggest support of these factors. Participant #2 (female) initially chose Factor 2 (for which they were loaded significantly) but finally opted for Factor 1. The participant stated that their amendment was because of the fact that they knew nothing about fossil fuels and its effects on everything. Therefore, there were no issues regarding any religion-related statements but rather with a statement pertaining to the influences of human activities.

Participant #4 (male) was loaded significantly for Factor 3, although they chose Factor 5. However, they gave a qualitative feedback in the interview during Phase Four that was mainly religious in nature. They explained various concepts in terms of biblical scripture:

‘Maybe if I have to preach a little bit: you remember, in the Bible, when God was destroying Sodom and Gomorrah – this is one story – two: the other one, Noah and the Ark. When God said to Noah: ‘‘I’m going to destroy the world, because of peoples’ lifestyle that is contrary with me, their God’’. People took Noah … there were those who were ignorant: ‘‘what is he building?’’ …’ (When illustrating people’s ignorance regarding climate change) (Participant #4; male)

‘We must understand that the climate … there’s nothing that in the time of maybe God’s servant like Abraham or those people, Isaac and Jacob, you name it: there’s nothing different about the claim of God to man concerning the climate, you see? But I can only say, the disturbance of the climate to the extent whereby the temperature is abnormal – is burning the crops – it’s not that God approve it that way. No, there must be a disturbance from the point of view from the world …’ (Participant #4; male)

‘The ending of the world has nothing to do with what you may believe in. The only guidelines of the ending of the world is through the Biblical text. It is there, it is written there, it is captured there, all along. So if you want to know whether it is ending – yes, it is ending, but this climate change is not part of it. The only part of it, the link to it is this so-called war – there will be war, there will be lawlessness, children will bring children forth and so on. Violent society, corruption, selfishness …’ (Participant #4; male)

Possible reasons for Participant #4 not choosing either Factor 2 or Factor 3, which are geared towards a religious worldview regarding climate change, are discussed in a later section, where further reference is made to the above statements in conjunction with the Q-sort data of Phase Three.

### Pertinent Q-sorts and their rankings

Table 3 illustrates the rankings of selected Q-sorts based on their link to the topic of climate change and religious beliefs.

**TABLE 3:** Q-sorts related to climate change and religious beliefs.

No.	Statement	Factor arrays				
		1	2	3	4	5
8	The climate is determined by God.	−2	3	3	0	−1
9	Climate is not punishment for the sins that people commit.	3	0	0	1	−1
1	The climate is a natural part of the world we just have to accept and live with.	0	3	−1	−1	1
6	Climate change is not a sign that the world is ending.	1	1	−1	1	−1
7	Natural disasters happen when nature wants to reshape itself.	2	1	−2	−1	0

Consensus between factors 2 and 3 on the theme of religious beliefs reaches only to statement 9, where Factor 2 agrees with statements 1, 6 and 7 and Factor 3 disagrees with these statements. Factor 3 disregards the ‘natural’ element of climate, whereas Factor 2 draws the link between God and nature previously discussed. This ‘naturalness’ of the climate and climate change stands opposite the view that climate change is a sign that the world is ending; if an occurrence is considered natural, it cannot simultaneously be considered an indication of the end of all things.

Although being seemingly more positive towards nature and climate, Factor 2 may be interpreted as being more deterministic or fatalistic towards the effects of climate change. This is mostly because of the high scoring of statement 1 – in fact, statement 1 scored higher in Factor 2 than in any other factor. This factor basically says, ‘climate change is a natural occurrence, therefore we cannot and will not do anything about it’ – adaptation seems to be regarded as futile. It is also noteworthy that Factor 2 denies human’s involvement in climate change, reinforcing the belief that the climate is controlled by God and is therefore impervious to human influence.

Statement 9, regarding punishment for mankind’s sins, did not load significantly for either factor. Factor 5 alone indicated an overall disagreement with the statement (-1), indicating the belief that climate change is indeed punishment.

Table 4 illustrates statements pertaining to ancestral beliefs and traditional healers.

**TABLE 4:** Q-sorts linked to ancestral beliefs and traditional healers.

No.	Statement	Factor arrays				
		1	2	3	4	5
10	Climate change is caused by the fighting of the ancestors.	−3	−3	−1	−2	−2
11	Traditional healers cause the climate to change.	−3	−3	0	−1	1
24	Educating people about climate change will anger the ancestors and cause bad luck.	−3	−2	−1	−2	−2

Statements 10 and 11 ranked lower for Factor 2 than for any other factors (-3, -3), indicating strong disagreement with the belief that ancestors and traditional healers can influence the climate. Statement 11, however, ranked higher with Factor 3 than any other factor (-1). This does not indicate agreement with the statement but may rather point to more of a willingness to accept the possibility of traditional healers playing a role in climate change.

Table 5 illustrates all statements directly related to beliefs.

**TABLE 5:** Q-sorts related to beliefs.

No.	Statement	Factor arrays				
		1	2	3	4	5
33	It is difficult to educate people about climate change because of their beliefs.	0	−1	0	−2	2
34	It is possible to change my beliefs when someone else tells me to.	−1	−2	−2	0	−2
35	In order to change our beliefs about the climate, we must sit down and discuss the matter.	2	1	1	1	1
36	My beliefs about the climate can change if I see in reality that things are different from what I believe.	2	2	2	0	2
37	My beliefs about the climate can change when I feel less vulnerable.	0	0	−2	0	0
38	I am open to change my beliefs because I learn new things all the time.	1	2	0	0	3
39	It is not possible to change my beliefs.	−1	−1	1	0	−2
40	The climate influences how people feel emotionally and that may influence their beliefs.	1	0	−1	−1	0

Factor 2 displays an openness to changing beliefs that are reflected in the statements above. For Factor 2, beliefs can be changed, and they should therefore pose no hindrance to education regarding climate change – in other words, their beliefs should not be a barrier to implementing climate change adaptation. Statement 36 ranked highest for this factor, emphasising that ‘seeing is believing’ and that telling someone to change their beliefs will have no impact (statement 34 ranked lower than any other factor).

Factor 3, however, exhibits some contradiction in terms of whether or not beliefs can be changed; they agree with both statements 39 and 36. This ambiguity lies at the heart of the stumbling block that religious beliefs pose to climate change adaptation. An explanation for this ambiguity might lie in the link between statements 34 and 39: statement 39 can be seen as implying that the change in belief is being enforced by external pressures, clearly supporting factor 3 in denying that beliefs can change when someone else tells them to. If they realise that their beliefs are untrue, they feel that they would be able to adapt and change those beliefs (statement 36 scored the highest for this factor).

## Discussion

During Phase Four, where participants were asked to identify the worldview they related to most, the majority of participants chose factors 1 and 4. Factors 2 and 3, which were discussed in the last section, were not chosen at all – the following section discusses the possible reasons for this.

Firstly, it is important to bear in mind that religious beliefs are often very prescriptive and specific, and elements like doctrine and subjective projection may underlie certain beliefs. They can also be either individually or communally held (Loubser 2013:72). It is for this reason that the formulation of certain Q-sort statements that may be regarded as being of a religious nature at face value (e.g. statement 8) may not coincide with a participant’s specific formulation of their religious beliefs, causing them to disagree with the statement altogether. This binary thinking stands in opposition to the evidential non-binary thinking exhibited throughout the research process, indicating a possible exception for religious beliefs. Beliefs entail more of what can be seen as disbelief – ‘on the one hand a system of beliefs which one accepts, and on the other, a series of systems of beliefs which one rejects’ (Rokeach 1956:228) – and this will result in participants rather discarding statements they do not agree with. This rigid belief–disbelief dualism seems to be especially prominent in thinking that involves deeper religious commitments. This does not, therefore, automatically exclude religiosity when formulating conceptions of climate and climate change – allowance should be made for the participant rather holding different religious beliefs. This was illustrated especially clearly in the case of Participant #4 in Phase Four of the research process. Participant #4 loaded significantly for Factor 3 but instead opted for Factor 5. This variance may be attributed to the fact that they explained their religious views extensively during the actual interview in Phase Four, citing reasons for their climate beliefs but seemed to disagree with the individual statements that comprise the Factor 3 narrative.

A further issue best illustrated by Participant #4 is that of the individual statements that comprise each narrative. Arguably the best way of determining participant worldviews is having them read through the options and make an intuitive choice, but because of the large volumes of information, this did not seem entirely possible. Instead, participants virtually analysed each worldview based on the individual statements, rather than choosing it based on an overall impression. This reflects a possible problem for participants to keep all the components of the narrative of their own identity under consideration at the intuitive level. As Lehrer (1973:121) almost poetically states: ‘The shifting sands of subjectivity shape and reshape the foothill paths of evidence that guide us to conclusions in the mountainous terrain of inquiry’. This inquiry can be into our own worldview, and the ‘evidence statements’ that we choose or discard when determining our own views often change (Lehrer 1973:121). Without the discussion of the different worldviews, and by default the different statements, the valuable qualitative input necessary for a study of this nature would not have been attained.

## Conclusion: Factor interpretations

Aside from ascertaining that religious beliefs do indeed influence climate change adaptation, this study produced one other main finding in the form of two distinct groups of religious participants. Firstly, Factor 2 participants are characterised by theological determinism; these participants view the climate as being a concept that lies within God’s realm, and therefore, people can have no influence on it. They do however acknowledge that climate influences people. This view can be detrimental to the cause for adapting to climate change, as it engenders a negligence that adversely affects the reciprocal relationship between humankind and nature. This view coincides with the idea of humankind’s dominion over nature, rather than stewardship, where humans can use and abuse nature in any way they choose (and consequently contribute to climate change). Still, there is the view that nature remains a creation of God that cannot be interfered with or changed. The belief in a sovereign God (who controls and determines all) relates to lower levels of concern over climate change, according to Peifer, Khalsa and Ecklund (2016:665). Ultimately, the willingness to change their beliefs, as expressed in the factor arrays, makes this group a prime candidate for accepting climate change adaptation education.

The second group, exemplified by Factor 3, rejects the ‘naturalness’ of climate and subsequently does not deify nature the way Factor 2 does. For these participants, climate change is understood in more concrete terms, and they choose to acknowledge humankind’s role in it. Adaptation here needs to be motivated intrinsically, perhaps making use of conceptual change methods of education.

### Recommendations and limitations

In conclusion, this study has achieved the overall aim of illustrating the influence that religious beliefs have when considering a person’s willingness or ability to adapt to climate change. As is almost always the case, further research on the topic is urged, with the distinction that the research is done mindful of the specific context it is conducted in. In other words, more research is required on this nature to determine other specific communities’ climate change and religious beliefs, which would ultimately engender an atmosphere of adaptation rather than mitigation. When working with people, understanding them is essential, especially in terms of climate change adaptation. This study has successfully illuminated the religious aspect of this sentiment. Incorporating such religious beliefs in climate change and climate change adaptation education may assist in obtaining deeper knowledge of a community’s needs and shed light on which practices are acceptable within their religion.

A clear limitation was anticipated for this study in terms of language, and this was addressed by involving researchers who are proficient in Tswana (home language for the majority of participants) and also making subsequent transcriptions and verbatim translations of these interviews.

## Footnotes

1 For a detailed discussion on Q-methodology and its steps, see: Stephenson, W. 1993/1994. Introduction to Q-Methodology. *Operant Subjectivity*, 17(1/2):1–13.

2 For a complete list of Q-statements (Phase Three), see Appendix 1.

3 For example: 01, participant number; GP, interviewer; 14:13, interview duration.

## Appendix 1: Q-sample

1. The climate is a natural part of the world we just have to accept and live with.
2. The climate is complicated.
3. The climate is unpredictable.
4. The climate is not changing.
5. There is something wrong with the climate.
6. Climate change is not a sign that the world is ending.
7. Natural disasters happen when nature wants to reshape itself.
8. The climate is determined by God.
9. Climate change is not punishment for the sins that people commit.
10. Climate change is caused by the fighting of the ancestors.
11. Traditional healers cause the climate to change.
12. The climate is affected by the behaviour of people.
13. Increasing population growth causes climate change.
14. Climate change is not caused by technology.
15. Climate change is related to the burning of fossil fuels and pollution.
16. The climate influences the growth of crops and the production of food.
17. People are trying to make money, that’s why they are damaging the environment.
18. The climate was not better when I was younger.
19. We can solve environmental problems by returning to the ways of the past.
20. The next generation will be influenced by our current behaviour towards nature.
21. We must act now to prevent the climate problems of the future.
22. Young people can help older people catch up with new knowledge about the climate.
23. We have the right to know about climate issues that affects us directly and indirectly.
24. Educating people about climate change will anger the ancestors and cause bad luck.
25. It is not the duty of the government to inform people about climate change.
26. We can address climate problems by drafting laws that protect the environment.
27. We can solve climate problems when we stand together and unite.
28. It is possible for humans to control the climate through technology.
29. Using sustainable technology is not good for the climate.
30. It is difficult to care about climate change because of economic pressures.
31. The climate does not play an important role in our lives.
32. We do not have to respect the environment.
33. It is difficult to educate people about climate change because of their beliefs.
34. It is possible to change my beliefs when someone else tells me to.
35. In order to change our beliefs about the climate, we must sit down and discuss the matter.
36. My beliefs can change if I see in reality that things are different from my beliefs.
37. My beliefs about the climate can change when mechanisms are in place to protect us.
38. I am open to change my beliefs, because I learn new things all the time.
39. It is not possible to change my beliefs.
40. The climate influences how people feel emotionally and that may cause changes in their beliefs.
